# Number theory, borderline dimension and extensive entropy in distributions of ranked data

**DOI:** 10.1371/journal.pone.0279448

**Published:** 2022-12-27

**Authors:** Carlos Velarde, Alberto Robledo

**Affiliations:** 1 Instituto de Investigaciones en Matemáticas Aplicadas y en Sistemas, Universidad Nacional Autónoma de México, Mexico City, Mexico; 2 Instituto de Física y Centro de Ciencias de la Complejidad, Universidad Nacional Autónoma de México, Mexico City, Mexico; Instituto Tecnologico de Monterrey, MEXICO

## Abstract

The consideration of an existing stochastic approach for the reproduction of ranked data pointed at a formal equivalence between its key mathematical expression and that for trajectories at the tangent bifurcation. This fact led to a nonlinear dynamical approach for rank distributions that shows similarities with universality classes in critical phenomena. The renormalization group (RG) fixed-point map f*(*x*) for a tangent bifurcation of arbitrary nonlinearity *z* > 1 has proved to be a powerful tool into which the formalism can be couched. The source distribution *P*(*N*) of the stochastic approach can be linked to f*(*x*) while the size-rank *N*(*k*) and frequency-rank *F*(*k*′) distributions are obtained, respectively, from the map trajectories *x*_*t*_ and the sums of its positions. We provide now an extension to Number Theory as we obtain from the trajectories *x*_*t*_ of f*(*x*) the numbers, or asymptotic approximations of them, for the Factorial, Natural, Prime and Fibonacci sets. A measure of the advance of these numbers towards infinity is given by sums of positions that represent their reciprocals. We specify rank distribution universality classes, already associated with real data, to these number sets. We find that the convergence of the series of number reciprocals occurs first at nonlinearity *z* = 2, that which corresponds to the classical Zipf law, and link this transition edge to the action of the attractor when it first reduces the fractal dimension of trajectory positions to zero. Furthermore, the search of logarithmic corrections common to borderline dimensions provides a link to the Prime numbers set. Finally, we find corroborating evidence of these logarithmic corrections from the analysis of large data sets for ranked earthquake magnitudes. The formalism links all types of ranked distributions to a generalized extensive entropy.

## Introduction

It is possible to evaluate complex information reordering numerical data into an integer index sequence of entries according to a certain criteria, i.e. by ranking them. This procedure has been in practice from immemorial times, and, looking back only to the last century, this exercise has yielded important empirical laws, such as those of Gutenberg and Richter for earthquakes [1, 2], Zipf for words in texts [3, 4], and Benford for digits in different lists of numerical data [5, 6]. The monotonic decay of ranked data often displays a conspicuous power law interval and a degree of universality that have puzzled many and prompted quests for finding underlying mechanisms [7]. A power law exponent often close to −1 together with the indistinguishability found in practice between ranking according to magnitude or frequency has led to a large body of processed data [8]. Nowadays, this topic is prominent amongst the complex systems community [7, 9].

Instead of fitting data with a specific distribution to infer or test the underlying physical mechanism responsible for the omnipresence of the classical Zipf law, we have pursued the view [10–16] that an understanding of the omnipresence of this type of rank distribution hints at a global structure similar to that which confers systems with many degrees of freedom the familiar macroscopic properties described by thermodynamics. That is, the quantities used in describing this empirical law obey expressions derived from principles like those for equilibrium statistical mechanics [10–16]. To obtain a global description for rank distributions it is assumed that real data samples can be reproduced statistically by considering that the numbers in a sample are random variable outcomes generated by a parent or source distribution [16, 17]. Then, the possible types of rank distributions can be obtained by considering parent distributions with all possible decay rates from logarithmic to exponential decay passing trough all possible power law decay rates [16, 17]. Furthermore, it was found that the rank distributions for all such parent distributions are equivalently obtainable from nonlinear iterated maps close to or at a tangent bifurcation, where now data samples are reproduced by their trajectories, including Zipf law [10–16]. That is, numerical values in data samples are equally generated by deterministic nonlinear dynamical low-dimensional systems. Additionally, a clear conceptual difference arises between magnitude and frequency rank distributions, the former is a quantile, while the latter is a cumulative distribution. These functions are inverses of each other and have the same power law exponent −1 for the Zipf class (sizes of cities obey the same power law as occurrences of words) [15]. It was also found that the reproduction of all classes of data satisfy a maximum entropy principle that leads to an extensive generalized entropy, valid under the important restriction that access to its configurational space is severely hindered to a point that the allowed configurational space has a vanishing measure [13, 14].

Here we develop further the approach for rank distributions based on nonlinear iterated maps near or at tangent bifurcations to reveal a global structure similar to that occurring in critical phenomena, or, in the same way as that with central limit theorems, where there are universality classes and borderline divergences. We uncover this structure by exhibiting links with Number Theory (in particular the Natural and the Prime numbers for the class that corresponds to the empirical Zipf law). The renormalization group (RG) fixed point map f*(*x*) for the tangent bifurcation (tangent at *x* = 0 with non linearity *z* > 1) plays a central role in our description [18], but also, for the first time, its extension to nonlinearity 0 ≤ *Z* = 2 − *z* ≤ 1 (when f*(*x*) shows a cusp at *x* = 0 or it is off tangency). Beside signs, we have: i) The trajectories of f*(*x*) initiated at *x* < −1 correspond to the size-rank distribution, while the sum of their positions (in a continuum limit the area under these trajectories) is the frequency-rank distribution, the inverse function of the size-rank distribution [15]. ii) The reciprocals (algebraic inverses) of these trajectories are trajectories initiated at 0 < *x* < 1 and they provide the values of uniformly-distributed probabilities from which the maximum entropy property of the rank functions is obtained [14]. This is described in the following Section 2. iii) The trajectories initiated at *x* = 1 (or at some other fixed number *x* > 1) are used to generate (in some cases asymptotically) number sets: Fibonacci (*z* = 1), Naturals (*z* = 2), Primes (*z* = 2 with a logarithmic correction) and Factorials (*z* → ∞). iv) The reciprocals of these trajectories are trajectories initiated at *x* > −1 and their sums define or relate to the Fibonacci *ζ* function (with *ζ* = 1), the Harmonic numbers, the Riemann *ζ* function (with *ζ* = 1) and the exponential function. The convergence or divergence of their infinite sums define two different behaviors of the trajectories of f*(*x*) according to whether the nonlinearity *z* is larger or smaller than *z* = 2. This is described in Section 3.

The action of the attractors of dissipative nonlinear systems is to reduce the position space available to trajectories, the configurational space in statistical-mechanical terms. The position space dimension is unity for maps on an interval. Chaotic attractors do not change the dimension of this space, multifractal attractors reduce the dimension below unity, and periodic attractors, like that at the tangent bifurcation, reduce the dimension to zero. The contraction dimension for f*(*x*) is *Z* = 2 − *z* when 1 < *z* < 2 and it vanishes for all *z* ≥ 2 [15]. Likewise, the series of reciprocals of the sets of Fibonacci and Factorial numbers converges and their f*(*x*) have *Z* = *z* = 1, while those series for the Natural and Prime numbers diverge, slowly (logarithmically) for the Naturals and very slowly (logarithm of logarithm) for the Primes. The latter case can be identified as a marginal case at which logarithmic corrections arise, as in critical phenomena [19]. This is described in Section 4. For the classical Gutenberg-Richter and Zipf laws we have *z* = 2 while the presence of logarithmic corrections are technically difficult to detect. We present numerical results, also in Section 4, for large earthquake data sets where we obtain a scaling property consistent with this borderline corrections. In the final Section 5 we summarize and discuss our results.

## Recall. Rank distributions via iterated maps at or near tangency

An upfront stochastic approach [17] to obtain theoretical size-rank functions *N*(*k*) considers samples for the magnitudes *N* of unspecified kinds of data to be represented by sets of random values generated by a parent or source distribution *P*(*N*). When *P*(*N*) is chosen to be the power law *P*(*N*) ≈ *N*^−*α*^, *α* > 1, one obtains [11, 17]

$$\begin{array}{c} N\left(k\right)=N_{\text{max}}\;{\text{exp}}_{\alpha }\left[-N_{\text{max}}^{\alpha -1}N^{-1}k\right], \end{array}$$ (1)

where *N*_max_ is the largest data value (with initial rank *k* = 0), N is the total number of data in the sample, and exp_*q*_(*x*) ≡ [1 + (1 − *q*)*x*]^1/1−*q*^ is the *q*-deformed exponential function. Moreover, when *N*_max_ → ∞, a pure power-law decay follows, *N*(*k*) ∝ *k*^1/1−*α*^, *α* > 1. And when *α* = 2 it takes the ‘classical’ Zipf form *N*(*k*) ∝ *k*^−1^.

Now, a very different situation is the (longtime established nonlinear dynamical) problem of determination of a functional-composition renormalization-group (RG) fixed-point map for a transition to chaos such as, for example, the tangent bifurcation [18, 20]. This is to find the function f*(*x*) (and the value of *γ*) that is the solution of f*(*x*) = γf*(*x*/*γ*), such that it also complies with the generic form for a map at tangency when *x* is small, f*(*x*) = *x* + *u*|*x*|^*z*^ + ⋯, *z* > 1. The solution is [18, 20]

$$\begin{array}{c} f^{*}\left(x\right)=x\;{\text{exp}}_{z}{\left(u|x\right|}^{z-1}\text{sgn}\left(x\right)),\;\gamma =2^{1/(1-z)}, \end{array}$$ (2)

a map with the scaling property, that reflects into the fact that all its trajectories obey the form [11]

$$\begin{array}{c} x_{t}=x_{0}\;{\text{exp}}_{z}\left(-\right|x_{0}{|}^{z-1}ut),\;x_{0}<0. \end{array}$$ (3)

**Fig 1:**
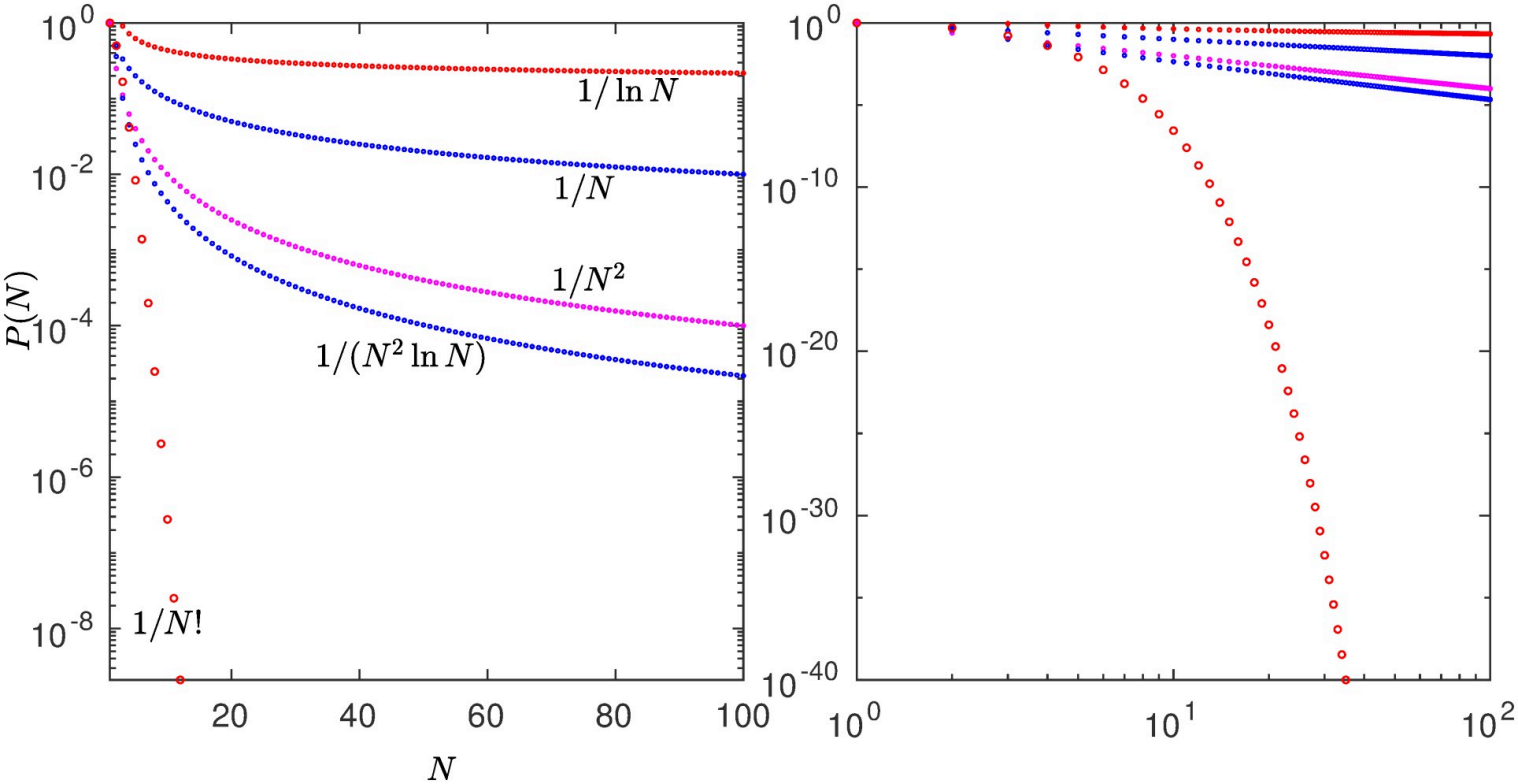
Different decay rates for the parent distribution *P*(*N*), left panel in semi-logarithmic scales, right panel in logarithmic scales.

Eqs (1) and (3) are identical, one transforms into the other through the equivalences *k* = *t*, *N*(*k*) = −*x*_*t*_, *N*_max_ = −*x*_0_, *α* = *z* and $N=u^{-1}$. Remarkably, size-rank distributions *N*(*k*) for all power-law exponents *α* can be reproduced by trajectories of the fixed-point map f*(*x*) [11]. And as it turns out also for *α* → ∞. More generally, *N*(*k*) for all source distributions *P*(*N*) can be obtained from trajectories of the map *x*′ = *x* + *u*/*P*(−*x*) under the same scheme [16]. In the continuum time limit the map becomes the differential equation *dx*_*t*_/*dt* = *u*/*P*(−*x*), that for the choice *P*(−*x*) = |*x*|^*z*^ its solution is the analytical expression in Eq (3) for the trajectories of f*(*x*). In Fig 1 we show different decay rates *z* = *α* for *P*(*N*) that will be useful for our discussion below. These are: A) factorial (exponential) decay, B) inverse square power decay with a further logarithmic correction decrement, C) inverse square power decay, D) inverse single power (hyperbolic) decay. Logarithmic decay is also shown in Fig 1. We have employed the specific iterated maps f*(*x*) that reproduce ranked data *N*(*k*) for these four decay rates as trajectories *x*_*t*_ with *N*_max_ = −*x*_0_. The *N*(*k*) are not normalized so that *N*_max_ is generally a large positive number and *x*_0_ << −1. In all cases trajectories start at the left of the point of tangency *x* = 0, for which all positions *x*_*t*_ < 0. We have reproduced real ranked data that match these choices: A) Gun ownerships [16], B) earthquakes (see below), C) California forest-fire areas [13] and USA city populations [16], D) infant mortality [16].

Therefore, the stochastic and the deterministic approaches are equivalent. This duality facilitates an explicit and quantitative distinction between size-rank *N*(*k*) —sizes of cities— and frequency-rank *F*(*k*′) —word frequencies— distributions, as the former appears as a trajectory while the latter is a sum of positions [15]. The frequency-rank distribution *F*(*k*′) turns out to be the functional inverse of *N*(*k*) [15]. The frequency-rank distribution is the complementary cumulative distribution of the parent distribution, while, technically, the size-rank distribution is not a distribution but a quantile (cut points dividing the range of a probability distribution into continuous intervals with equal probabilities). See Fig 2 in Ref. [15].

**Fig 2:**
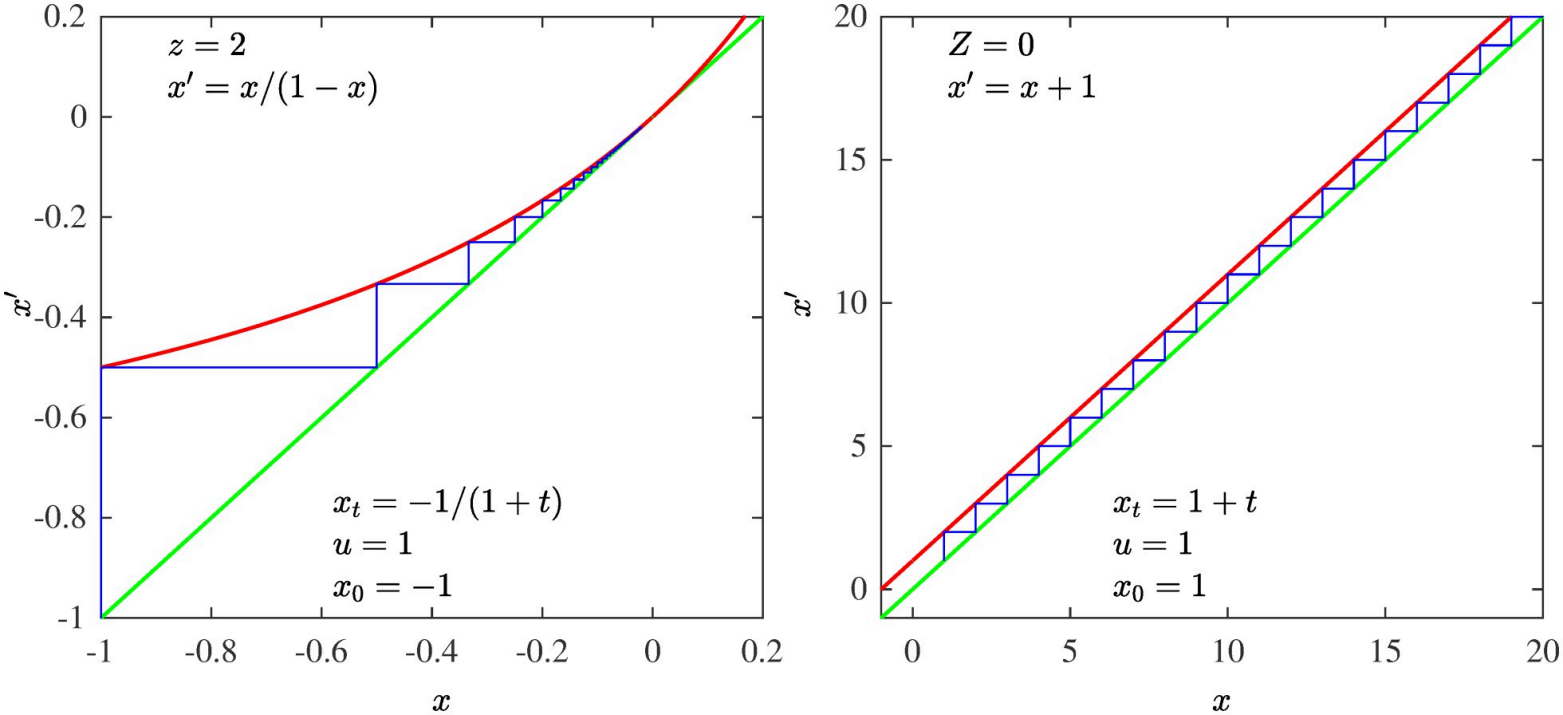
Natural numbers. Left panel: In red the map *x*′ = *x*/(1 − *x*) (f*(*x*) with *z* = 2 and *u* = 1). In blue the trajectory *x*_*t*_ = −1/(1 + *t*) initiated at *x*_0_ = −1. Its positions are (minus) the reciprocals of Natural numbers. Right panel: In red the map *x*′ = *x* + 1 (f*(*x*) with *Z* = 0 and *u* = 1). In blue the trajectory *x*_*t*_ = 1 + *t* initiated at *x*_0_ = 1. Its positions are the Natural numbers. Identity lines in green.

There is another significant property related to this topic that can be obtained from the map at tangency. The reciprocals of *N*(*k*) provide uniformly-distributed probabilities $p_{i}^{\left(k\right)}=p^{\left(k\right)}=1/N\left(k\right),i=0,...,k$ (one set of *k* + 1 equal probabilities for each value of *k*, 0 ≤ *k* ≤ *k*_max_), that lead to extensive *q*-deformed entropies where system size is measured by sample size $k_{\text{max}}=N$ [13, 14]. The expression for the *q*-deformed entropy, or Tsallis entropy, is

$$\begin{array}{c} S_{2-q}=\sum_{i=0}^{k}p_{i}\;{\text{ln}}_{2-q}\;p_{i}^{-1}, \end{array}$$ (4)

where the *q*-logarithm ln_*q*_(*x*) ≡ [*x*^1−*q*^ − 1]/(1 − *q*) is the functional inverse of exp_*q*_(*x*). The extensivity of the entropy in Eq (4) can be corroborated when considering that the system size is given by *k*_max_, the total number of data (minus one) in the sample of magnitudes *N*(*k*) We have $S_{2-q}\left(k_{\text{max}}\right)={\text{ln}}_{2-q}\left[p^{\left(k_{\text{max}}\right)}/p^{\left(k=0\right)}\right]∼N,q=z=\alpha$ [13, 14]. The numbers *N*(*k*), we recall, were obtained from trajectories, with *x*_*t*_ < −1, *t* = 0, 1, …, *t*_max_, from the *x* < 0 branch of the map. Therefore the probabilities *p*^(*k*)^ can be obtained as trajectories, with 0 < *x*_*t*_ < 1, *t* = 0, 1, …, *t*_max_, *t*_max_ = *k*_max_, from the *x* > 0 branch of the map [13, 14].

Finally, another noteworthy use of the nonlinear dynamical analogue for rank distributions is that the necessary consideration of the finite size effect of real data is rightly and promptly resolved by taking the matching map off tangency [16].

## Universality classes and number theory

We have made use of trajectories from the left branch *x* < 0 of maps at tangency (particularly f*(*x*)) with *x*_0_ < −1 and also of their reciprocals in the right branch *x* > 0 with *x*_0_ < 1. Large ranked data sets *N*(*k*) headed by large leading magnitude *N*_max_ are represented by trajectories initiated far into the left branch of the map, so that the associated sets of (uniform) probabilities *p*^(*k*)^ = 1/*N*(*k*) (one set for each *k*) are represented by trajectories initiated close to *x* = 0 in the right branch *x* > 0. We now make use of the remaining two map regions, the right branch *x* > 0 with trajectories starting from *x*_0_ ≥ 1 and running towards *x*_*t*_ → ∞, and their reciprocals in the left branch *x* < 0 starting from *x*_0_ ≥ −1 and running towards *x*_*t*_ → 0. Trajectories in the right branch, starting and running now with *x*_*t*_ ≥ 1, *t* = 0, 1, …, can be used to generate the most renowned sets of numbers (or increasingly better approximations of them). Fibonacci numbers when *z* = 1, Natural numbers when *z* = 2, Prime numbers when *z* = 2 with logarithmic corrections, Factorial numbers when *z* → ∞. In turn, the negative reciprocal of these numbers appear as trajectories from the *x* < 0 branch of the map with *x*_*t*_ > −1, *t* = 0, …, ∞. The series formed by these reciprocals converge for *z* > 2 but diverge for *z* ≤ 2, in fact the borderline for divergence manifests as *z* = 2 with logarithmic corrections, related to the known bounds for the Prime numbers and the very slow divergence of the sum of their reciprocals. This is reminiscent of borderline dimensionality and its logarithmic corrections in critical phenomena. A note about notation: Besides sign and a constant factor (often unity), the trajectory positions derived from the fixed-point map f*(*x*) in the right regions *x* > 0 are reciprocals of those in the left region *x* < 0, and vice versa, and these positions are given by the deformed exponential expression in Eq (3). We recall the algebraic inverse property 1/exp_*q*_(*x*) = exp_*Q*_(−*x*), *Q* = 2 − *q*, so that we use the notation *q* = *α* = *z* for *x* < 0 and *Q* = *α*′ = *Z* = 2 − *q* = 2 − *α* = 2 − *z* for *x* > 0.

### Natural numbers

The map that generates iteratively the natural numbers is very simple, it is the straight line, *x*′ = *x* + 1, parallel to the diagonal *x*′ = *x*. With the initial condition *x*_0_ = 1 this map produces the trajectory *x*_*t*_ = 1 + *t*. It is a (linear) particular case of the RG map f*(*x*) with *Z* = 0 and *u* = 1. The map for the reciprocals of the natural numbers is *x*′ = *x*(1 − *x*)^−1^. With the initial condition *x*_0_ = −1 we have *x*_*t*_ = −(1 + *t*)^−1^. The RG map f*(*x*) with *z* = 2 and *u* = 1. The harmonic numbers are obtained by the sum of positions *H*_*n*_ = −∑_*t*_ *x*_*t*_ ∼ ln *n* that diverges as *n* → ∞. See Fig 2.

### Prime numbers

**Fig 3:**
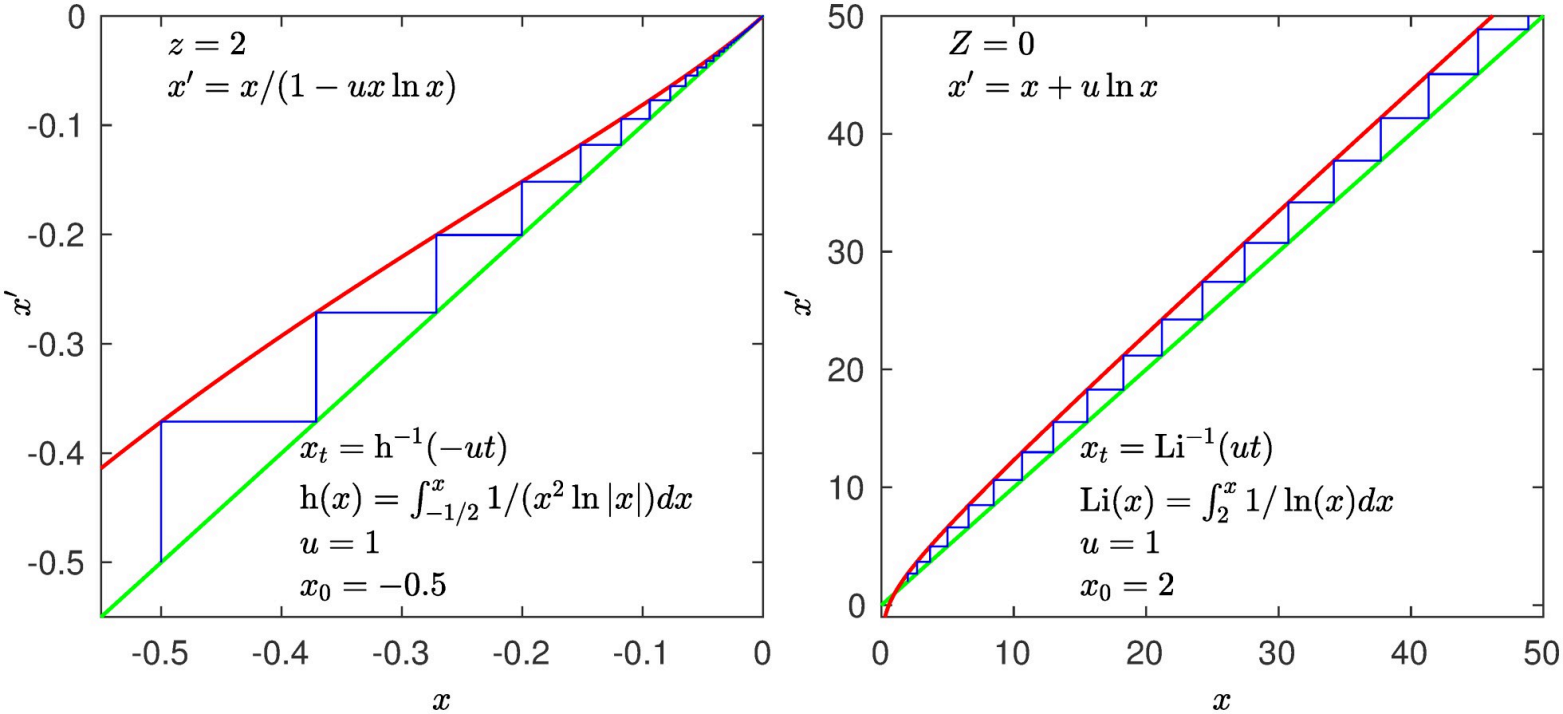
Prime numbers. Left panel: In red the map *x*′ = *x*/(1 − *x* ln *x*) (f*(*x*) = *x* exp_*z*_(*u*|*x*|^*z*−1^ ln *x*) with *z* = 2 and *u* = 1). In blue the trajectory $x_{t}=h^{-1}\left(-t\right),h\left(x\right)=\int_{-1/2}^{x}dx(1/x^{2}\;\text{ln}\;|x|)$ initiated at *x*_0_ = −1/2. Its positions approximate asymptotically (minus) the reciprocals of Prime numbers. Right panel: In red the map *x*′ = *x* + ln *x* (f*(*x*) = *x* exp_*Z*_(*u*|*x*|^*Z*−1^ ln *x*) with *Z* = 0 and *u* = 1). In blue the trajectory $x_{t}={\text{Li}}^{-1}\left(t\right),\text{Li}\left(x\right)=\int_{2}^{x}dx1/\text{ln}\;\left|x\right|$ initiated at *x*_0_ = 2. Its positions approximate asymptotically the Prime numbers. Identity lines in green.

The (logarithmic integral) upper bound for the number of Primes Π(*t*) up to the Natural number *t* can be used to define a map with a trajectory that approximates asymptotically the Prime numbers *p*_*t*_. The logarithmic integral function is $\text{Li}\left(x\right)\equiv \int_{2}^{x}1/\text{ln}\left(x\right)dx$, and the number of Primes in [2, *x*] is $\Pi \left(x\right)=\text{Li}\left(x\right)+O\left(\sqrt{x}\;\text{ln}\;x\right)$ [21]. A map that generates iteratively numbers that approximate asymptotically the Prime numbers *p*_*t*_ is *x*′ = *x* + ln *x*, *x* > 0. With initial condition *x*_0_ = 2 this produces a trajectory that approximates asymptotically the inverse of the Li function. The map can be expressed as f*(*x*) with a logarithmic factor in the argument of the deformed exponential, this is *x*′ = *x* exp_*Z*_(*ux*^*Z*−1^ ln *x*) with *Z* = 0 and *u* = 1. The corresponding map that approximates the reciprocals of the Prime numbers is *x*′ = *x*/(1 − *x* ln |*x*|), *x* < 0. The trajectory with initial condition *x*_0_ = −1/2 approximates asymptotically *x*_*t*_ = *h*^−1^(−*t*) where $h\left(x\right)\equiv \int_{-1/2}^{x}1/\left(x^{2}\;\text{ln}\;x\right)dx$. Also for this case the map can be expressed as f*(*x*) with a logarithmic factor in the argument of the deformed exponential, *x*′ = *x* exp_*z*_(*ux*^*z*−1^ ln *x*) with *z* = 2 and *u* = 1. The sum of Prime reciprocals $\sum_{t}p_{t}^{-1}∼\text{ln}\left(\text{ln}\;x\right)$ diverges (as known, very slowly,) when *n* → ∞ as it does the sum of positions *x*_*t*_. See Fig 3.

### Fibonacci numbers

**Fig 4:**
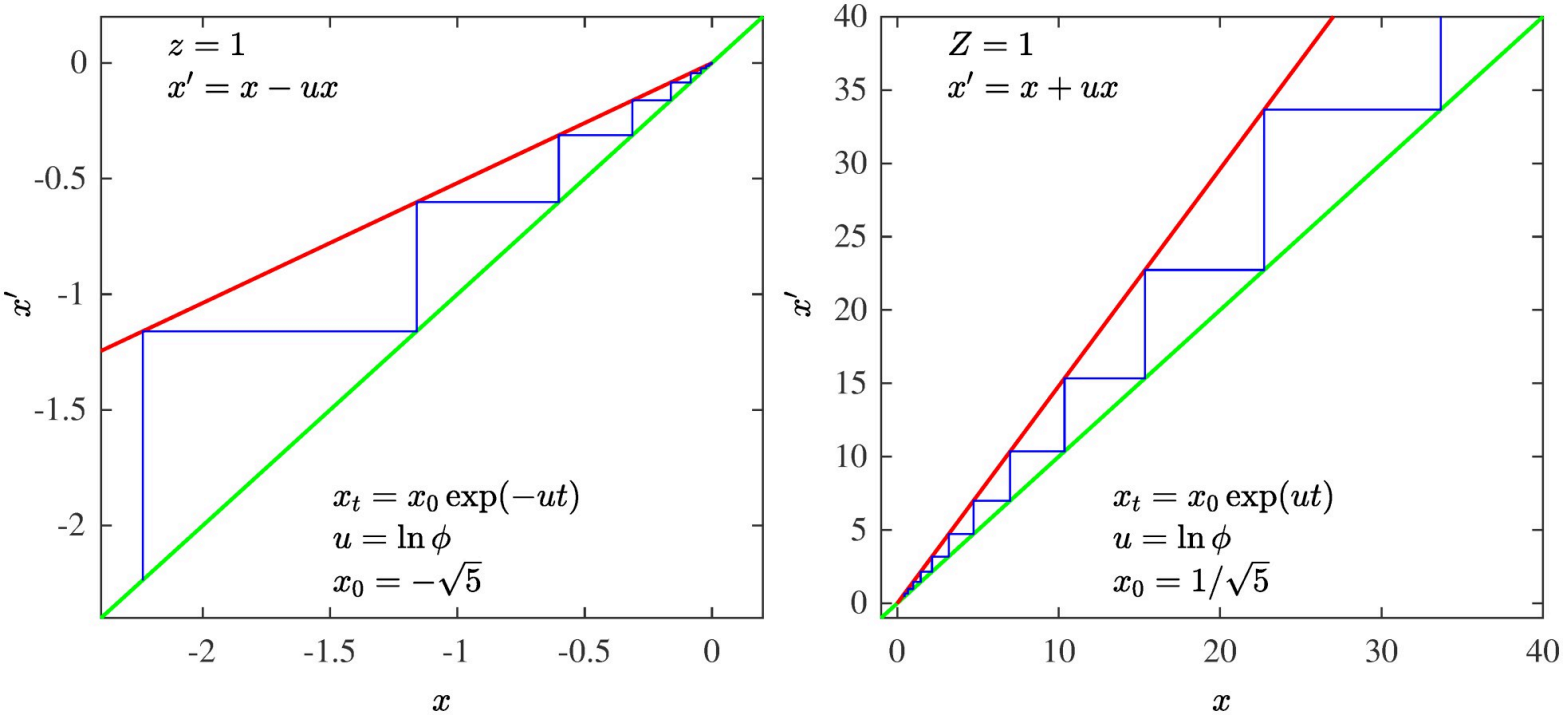
Fibonacci numbers. Left panel: In red the map *x*′ = *x* − *ux* (f*(*x*) with *z* = 1 and *u* = ln *ϕ* with $\phi =(1+\sqrt{5})/2$). In blue the trajectory *x*_*t*_ = *x*_0_ exp(−*ut*) initiated at $x_{0}=-\sqrt{5}$. Its positions approximate asymptotically (minus) the reciprocals of the Fibonacci numbers. Right panel: In red the map *x*′ = *x* + *ux* (f*(*x*) with *Z* = 1 and *u* = ln *ϕ*). In blue the trajectory *x*_*t*_ = *x*_0_ exp(*ut*) initiated at $x_{0}=1/\sqrt{5}$. Its positions approximate asymptotically the Fibonacci numbers. Identity lines in green.

The Fibonacci numbers *F*_*t*_, *t* = 1, …, are given by the Binet formula $F_{t}=\left(\phi^{t}-\psi^{t}\right)/\left(\phi -\psi \right),\phi =\left(1+\sqrt{5}\right)/2>0,\psi =\left(1-\sqrt{5}\right)/2<0$ [22], so that elimination of the term *ψ*^*t*^ in the numerator of this formula yields an exponential approximation for *F*_*t*_ for large *t*. A linear map that generates iteratively numbers that approximate asymptotically the Fibonacci numbers *F*_*t*_ is *x*′ = *x* + (ln *ϕ*)*x*, *x* > 0. This map produces a trajectory that approximates asymptotically the Fibonacci numbers as *x*_*t*_ = *x*_0_ exp((ln *ϕ*)*t*) with $x_{0}=1/\sqrt{5}$. The map can be expressed as f*(*x*) with *Z* = 1 and *u* = ln *ϕ*. The linear map for the (negative) reciprocals numbers is *x*′ = *x* − (ln *ϕ*)*x*, *x* < 0, as its trajectory *x*_*t*_ = *x*_0_ exp(−*t*) with $x_{0}=-\sqrt{5}$ approximates asymptotically the (negative of the) reciprocals of the Fibonacci numbers. The map can be expressed as f*(*x*) with *z* = 1 and *u* = ln *ϕ*. The sum of Fibonacci reciprocals $\sum_{t}F_{t}^{-1}$ converges as *t* → ∞ as it does the sum of positions *x*_*t*_. See Fig 4.

### Factorial numbers

**Fig 5:**
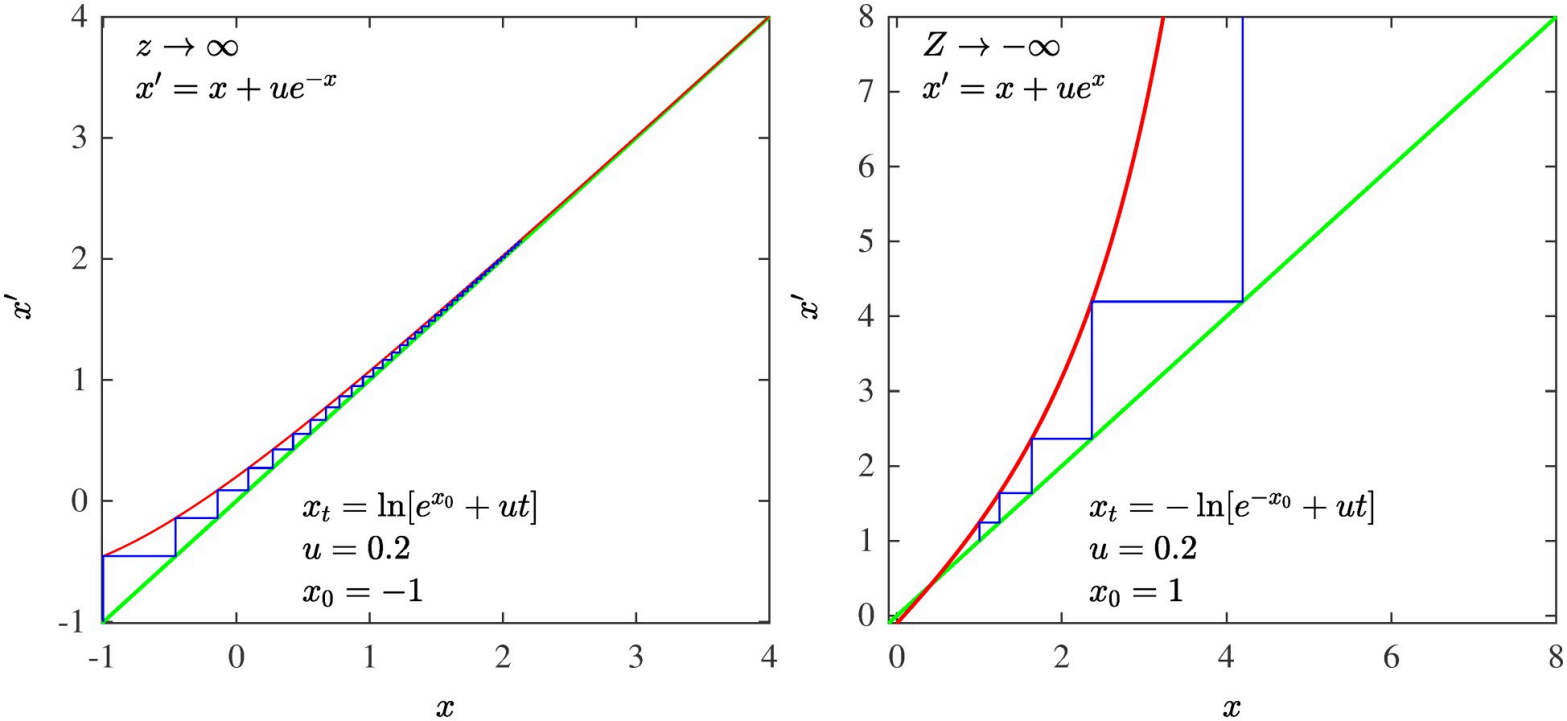
Factorial numbers. Left panel: In red the map *x*′ = *x* + *u* exp(−*x*) (f*(*x*) with *z* = →∞ and *u* = 0.1). In blue the trajectory *x*_*t*_ = ln[exp *x*_0_ + *ut*] initiated at *x*_0_ = −1. Its positions approximate asymptotically (minus) the reciprocals of the Factorial numbers. Right panel: In red the map *x*′ = *x* + *u* exp *x* (f*(*x*) with *Z* → −∞, 0 ≤ *u* ≤ 1). In blue the trajectory *x*_*t*_ = −ln[exp(−*x*_0_) + *ut*] initiated at *x*_0_ = 1. Its positions approximate asymptotically the Factorial numbers. Identity lines in green.

The Stirling approximation $t!\approx \sqrt{(}{2\pi t)\left(t/e\right)}^{t}$ for the factorial numbers *t*! can be used to define a map to generate iteratively asymptotic approximations to these numbers as *t* → ∞. Since here we are only interested in showing the general scheme we adopt a very crude approximation. To this purpose we choose the map *x*′ = *x* + *u* exp(*x*), that produces trajectories that approximate asymptotically (though slowly) the factorial numbers as *x*_*t*_ = − ln[exp(−*x*_0_) + *ut*], *x*_0_ = 1. This correponds to the fixed-point map f*(*x*) in the limit *Z* → −∞. The map to be used for the (negative) reciprocals of the factorial numbers is *x*′ = *x* + *u* exp(−*x*), as its trajectory *x*_*t*_ = ln[exp(*x*_0_) + *ut*], *x*_0_ = −1 approximates asymptotically the (negative) of the reciprocals of the factorial numbers. The map can be expressed as f*(*x*) with *z* → ∞. The sum of the factorial reciprocals ∑_*t*_(*t*!)^−1^ converges to the irrational number *e* as *t* → ∞ as it does the sum of positions *x*_*t*_. See Fig 5. Interestingly, the ordinary exponential (and its inverse the ordinary logarithmic) behavior is obtained by both the maps with nonlinearity *z* = 1 (and *Z* = 1) and *z* → ∞ (and *Z* → −∞). Other *q*-exponential (and its functional inverse *q*-logarithmic) behavior (asymptotic power law behavior) occurs for 1 < *q* = *α* = *z* < ∞.

In addition to the reproduction of the size-rank distributions *N*(*k*) of real data cases, the four different decay rates (A, B, C and D) for the source distribution *P*(*N*) in Fig 1 described above can be linked each to a well-known infinite set of numbers via the RG fixed-point map f*(*x*) for the tangent bifurcation. The nonlinear maps have each a different tangency feature, when *z* = 1 the map crosses the identity line, for *z* = 2 the map is tangent, and it is regular with nonzero second derivative or curvature *u* > 0, in the limit *α* = *z* → ∞ the tangency point shifts to infinity [16]. So, this is evidence that ranked data appear to belong to universality classes to be distinguished by the above values for *α* = *z*. The infinite sets of number theory help characterize the classes of rank functions but they also provide a statistical-mechanical insight. A canonical partition function is the sum of terms formed by two factors, numbers of configurations *W*_*k*_ and statistical weights *ω*_*k*_,

$$\begin{array}{c} Z_{k_{\text{max}}}=\sum_{k=0}^{k_{\text{max}}}W_{k}\omega_{k}, \end{array}$$ (5)

the growth of the numbers of configurations with the number of degrees of freedom (where here the rank *k* or the iteration time *t* plays the role of energy in a thermal system) is compensated by the decay of the statistical weights. The *W*_*k*_ can be identified with the *N*(*k*) or with the magnitude of the numbers in number theory sets, while the weights *ω*_*k*_ can be recognized as the uniform probabilities *p*_*k*_ or as the reciprocals of the numbers in the sets. The product *W*_*k*_*ω*_*k*_ is of order unity for all sizes of the system, here $k_{\text{max}}=N$, including the thermodynamic limit of infinite size, besides factors they are reciprocals of each other. This feature ensures the extensivity of the thermodynamic potential. The ordinary (Boltzmann-Gibbs) case is represented by the decay rate A, the Factorial numbers and their reciprocals. In statistical-mechanical terms decay rate cases B and C correspond to *q*-statistics with *q* = 2, the Natural and Prime numbers and their reciprocals. The ordinary case is recovered for the decay rate D, the Fibonacci numbers and their reciprocals, exponential growth and decay for *W*_*k*_ and *ω*_*k*_, respectively, reappears as the map intersects the identity line. In the neighborhood of the intersection the map approximates a straight line and *q* = 1.

## Borderline dimensionality

As we have seen the expressions for the size-rank functions *N*(*k*) and their equivalent map trajectories *x*_*t*_ obtained from f*(*x*) are *q*-deformed exponentials. Also, their functional inverses, the frequency-rank distributions *F*(*k*′) and their equivalent sums of trajectory positions are *q*-deformed logarithms. When *q* = *α* = *z* = 1 the ordinary exponential (*N*(*k*)) and its functional inverse the ordinary logarithm (*F*(*k*′)) are as different as they can be, separated by all power-law decay functions. When *q* = *α* = *z* grows from unity the *q*-deformed exponential and logarithmic functions develop a closer resemblance as they both acquire power-law decay. When the value *q* = *α* = *z* = 2 is reached the power-law decay for both functions becomes identical (explaining the common referral as Zipf law for both *N*(*k*) and *F*(*k*′)). There, *Q* = *α*′ = *Z* vanishes. The number *Q* = *α*′ = *Z* = 2 − *q* = 2 − *α* = 2 − *z*, with *q* = *α* = *z* ≤ 2 has been shown to represent a ‘contraction’ dimension, an index that quantifies the reduction of phase space exerted by an attractor [13, 14]. For a chaotic attractor *Q* = *α*′ = *Z* = 1, for a multifractal attractor *Q* = *α*′ = *Z* < 1, and for a periodic attractor (including a tangency point) *Q* = *α*′ = *Z* = 0. Thus, for all *q* = *α* = *z* > 2 the contraction dimension must remain zero. According to our formalism Zipf law appears at a borderline dimension similar to those in critical phenomena, or in the central limit theorems when the convergence or divergence of a second moment of symmetrical distributions leads to the normal distribution or to the Levy distributions, respectively. Interestingly, as we shall see, this edge appears to be represented by the set of Prime numbers.

**Fig 6:**
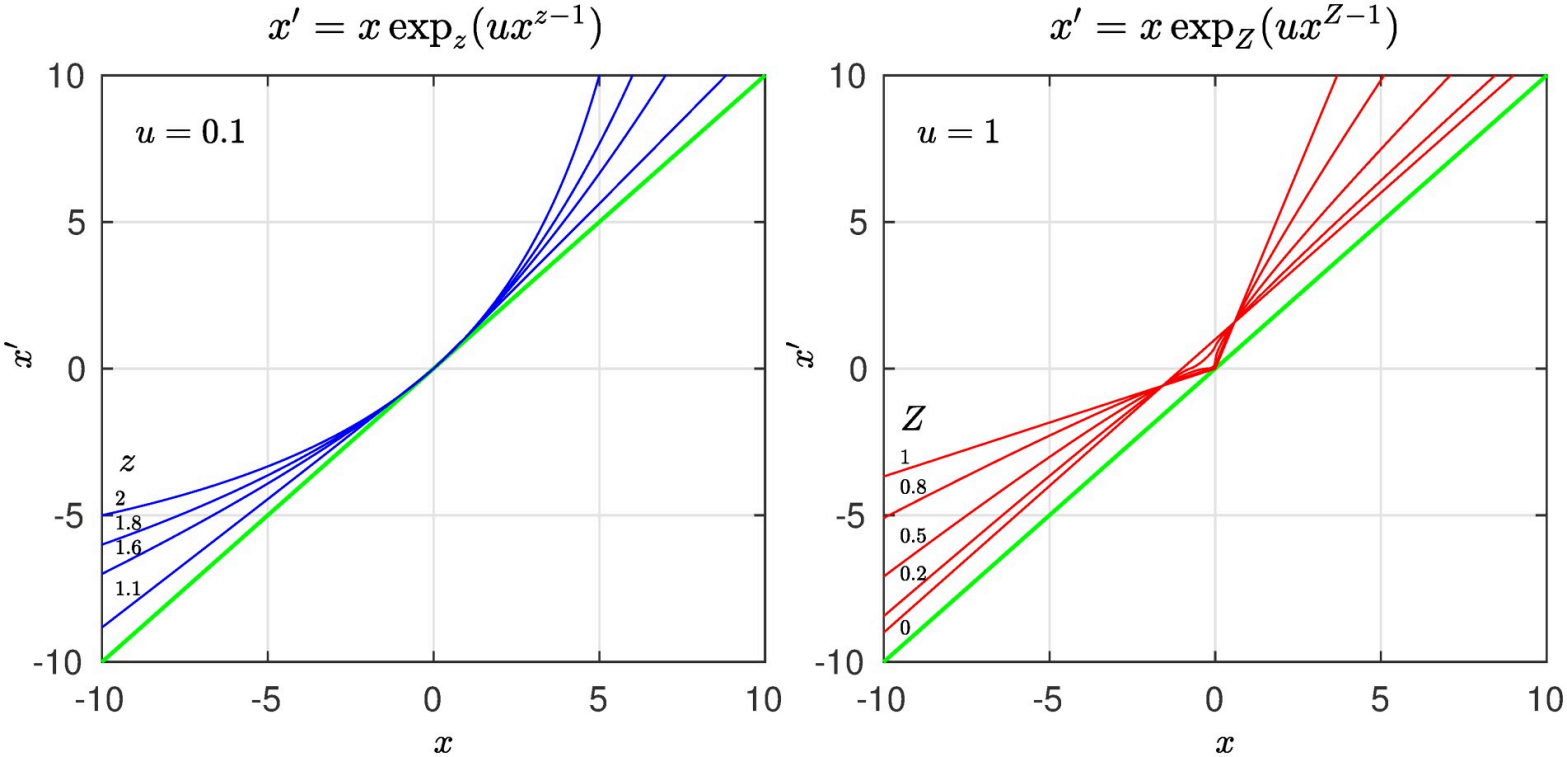
Fixed-point map f*(*x*). Left panel: The fixed-point map f*(*x*) for several values of the nonlinearity *z* > 1, exhibiting tangency at *x* = 0. Right panel: The fixed-point map f*(*x*) for several values of the nonlinearity *Z* < 1, exhibiting a cusp at *x* = 0 and then gradual separation from the identity line.

In Fig 6 we show the fixed-point map f*(*x*) for various values of *z*. In the left panel we observe tangency with the identity line when *z* > 1, when *z* ∼ 1 the map is very flat but as *z* grows acquires a more visible convexity, the special value *z* = 2 is included. The sums of trajectory positions *x*_*t*_ initiated with *x*_0_ < 0 diverge when *z* ≤ 2 but converge when *z* > 2. The case *z* = 2 with *u* = 1, *x*_0_ = −1 is illustrated by the (negative) Harmonic numbers. In the right panel we observe a cusp instead of tangency as well as separation from tangency when the nonlinearity is given values *Z* = 2 − *z* < 1 that correspond to the reciprocal *Q*-exponential. Two cases are noteworthy, the straight line cusp *Z* = 1 associated with the Fibonacci numbers and the line parallel to the identity line associated with the Natural numbers *Z* = 0.

Logarithmic corrections are characteristic of marginal scenarios. We have noticed that when *q* = 2 the limiting value for power-law decay is reached for convergence of the series of reciprocals of the number sets. When *q* = 2 without logarithmic correction the series is divergent and partial sums define the harmonics grow slowly as ln(*n*). This corresponds to the Natural Numbers (obtained with *Q* = 2 − *q* = 0). With the logarithmic correction the series is still divergent and the partial sums are made (approximately) of Prime number reciprocals. These sums grow even more slowly as ln(ln(*n*)). For simplicity we have placed a logarithmic correction into the decay of the parent distribution *P*(*N*) when *α* = *q* = 2 of the form *P*(*N*) ∼ 1/(*N*^2^ ln *N*). Besides a factor this is equivalent to the more general form *P*(*N*) ∼ 1/(*N*^2^ ln *N*^*β*^), *β* ≠ 1. Likewise, we introduced a logarithmic correction into the deformed exponential argument for f*(*x*), i.e. f*(*x*) = *x* exp_*z*_(*ux*^*z* − 1^ ln *x*). This argument would only be rescaled in the more general form f*(*x*) = *x* exp_*z*_(*ux*^*z*−1^ ln *x*^*γ*^), *γ* ≠ 1. Significantly, all the trajectories generated by the fixed-point map f*(*x*) for any value of the deformation *q* = *α* = *z* are of the form in Eq (3), *x*_*t*_ = *x*_0_ exp_*z*_(−|*x*_0_|^*z*−1^*ut*). Therefore any pair of trajectories obtained from f*(*x*), say for the same *q* = *α* = *z*, and possibly different number of iterations or different *u*, can be transformed into each other by adjusting the initial condition *x*_0_ and the parameter *u* (the map curvature for *z* = 2). That is, all trajectories are related to each other via simple rescaling. For example, a trajectory made of *t*_max_ iterations initiated at *x*_0_ in a fixed-point map f*(*x*) with given *z* and *u* can be transformed into another trajectory with the same number of iterations *t*_max_ initiated at $x_{0}^{\prime }$ in a map f*(*x*) with same *z* and $u^{\prime }={\left(x_{0}^{\prime }/x_{0}\right)}^{1-z}u$ simply by rescaling the former by a factor $T=x_{0}^{\prime }/x_{0}$. We use this property when considering logarithmic corrections.

**Fig 7:**
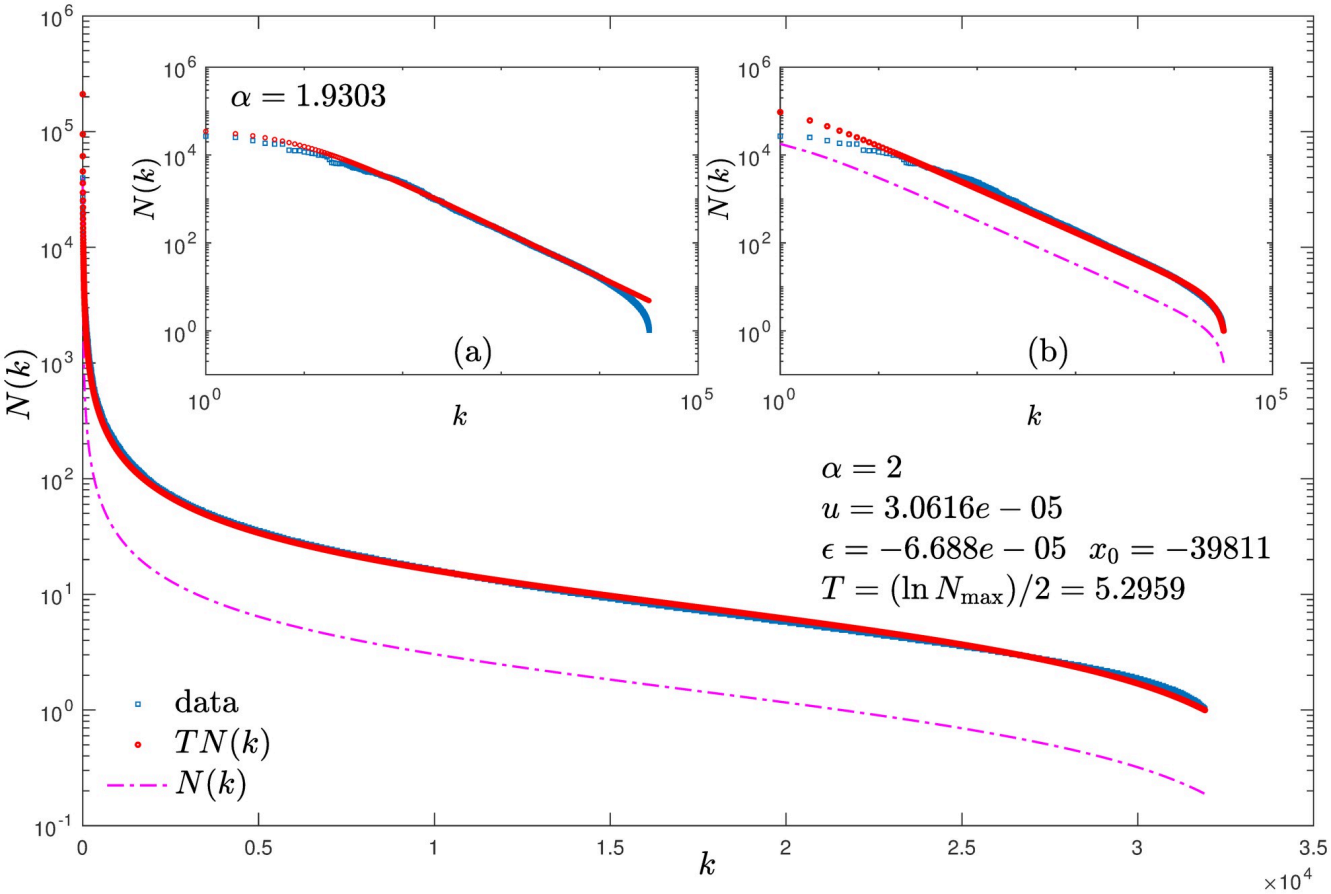
Ranked earthquake magnitudes combining California data [23] for the two years 2015 and 2017 shown in blue. Inset (a) data is fitted with a trajectory from Eq (3) with *q* = *α* = *z* = 1.9303 shown in red in logarithmic scales. Main panel: Magenta dashed-dotted curve shows a trajectory from Eq (3) with *q* = *α* = *z* = 2 and a small shift from tangency *ϵ* = 6.688 × 10^−5^ in semi-logarithmic scales. A vertical shift of a distance *T* = (ln *N*_max_)/2 = 5.2959 results in the red curve that fits the data in blue quantitatively. Inset (b) Same as main panel but in logarithmic scales.

Detection of logarithmic corrections in real ranked data sets that are known to obey Zipf law would require sufficiently large entries so that the logarithm of the magnitudes or frequencies manifest quantitatively. We have considered before California earthquake data [23] to exhibit the relationship between size-rank *N*(*k*) and frequency-rank *F*(*k*′) already mentioned [15]. We make use again of these data sets [23] to probe the presence of logarithmic corrections. In Fig 7 we show ranked magnitudes combining data for the two years 2015 and 2017 to obtain a data set with more than thirty thousand entries. The data is shown in blue in the figure and in its two insets. Fitting this data with a trajectory from Eq (3) as shown in red in inset (a) of Fig 7 in logarithmic scales yields *q* = *α* = *z* = 1.9303. Then we plot Eq (1), or equivalently, Eq (3), by keeping *q* = *α* = *z* = 2 as it corresponds with our theoretical Zipf law value and fixing *N*_max_ = 39, 811 and $N=u^{-1}=31,919$ in accordance with the data. Then we take the map off tangency a small amount *ϵ* = 6.68 × 10^−5^ (since the effect of finite size of real data for large rank is obtained by taking the matching map off tangency [16]). The result is shown by the magenta dash-dot curve in Fig 7 in semi-logarithmic scales and also in inset (b) of Fig 7 in logarithmic scales. As it can be observed, the curves runs mostly parallel to the data but way down below it. We now rescale this theoretical result, a map f*(*x*) trajectory for *z* = 2, by a scale factor *T* as described above, a shift in the logarithmic scales in Fig 7, and obtain, the red curves in Fig 7 and in its inset (b), a quantitative match with the data. The value we used for the scale factor is *T* = ln *N*_max_/2 = 5.2959 that falls within the logarithmic correction considered *N* → *N* ln *N*^*β*^, *β* = 1/2. This is our preliminary exploration of the possible presence of logarithmic corrections associated with borderline contraction dimension in our formalism with application to rank functions.

Via its scaling features RG universality provides direct answers about relevant properties and behaviors of complex systems. Otherwise these solutions may be reached through long and often cumbersome analysis that demands specific knowledge, mechanisms and facts about the system or problem under consideration. We exemplified this circumstance for the particular case of earthquake magnitudes.

## Summary and discussion

We have described a general theoretical formalism for rank distributions, both size-rank and frequency-rank, based on a well-known area of nonlinear dynamics, that of the tangent bifurcation in simple low-dimensional iterated maps. The main quantity in the scheme is the functional composition renormalization-group (RG) fixed-point map f*(*x*), first derived by Hu and Rudnick. This map displays a closed-form analytical expression involving a deformed exponential function exp_*z*_(*x*) with arbitrary nonlinearity *z* > 1 [10–16]. The deterministic formalism is equivalent [16] to a known stochastic approach [17] in which the entries of data samples (to be ranked) are random variables generated by a source or parent distribution *P*(*N*). Our approach reveals universality classes into which rank distributions can be clearly assigned [16], and clarifies the formal differences between magnitude and frequencies often grouped together in the Zipf law literature [15]. We have added a number theoretical aspect to our approach, with reference to Factorial, Natural, Prime and Fibonacci numbers, that clarifies further the nature of the mentioned universality classes. We have indicated the presence of borderline behavior similar to the borderline dimension in critical phenomena and borderline modification in central limit distributions. Lastly, we have made explicit a generalized statistical-mechanical structure in the formalism.

The decay rate *α* of the source distribution *P*(*N*) ∼ *N*^−*α*^ (c.f. Fig 1) translates into the degree of nonlinearity *z* of the fixed-point map f*(*x*) ∼ *x* + *u*|*x*|^*z*^ at tangency (c.f. Fig 6). The value of *α* = *z* marks the universality class. The derivation of f*(*x*) from *P*(*N*) is detailed in Ref. [16]. The analytical closed-form expressions for f*(*x*), Eq (2), and for its trajectories, Eq (3), essentially *q*-exponentials, facilitates visualization of the structure of the formalism. The algebraic inverses of trajectories link the values of the size-rank distribution *N*(*k*) with their probabilities *p*_*k*_, but also link the elements of the number sets (Factorials, Naturals, Primes, Fibonacci) to their reciprocals. The functional inverses of trajectories, sums of position reciprocals, link the size-rank distribution *N*(*k*) with the frequency-rank distribution *F*(*k*′), the quantile with the cumulative distribution, but also link the infinite number sets with the series that measures and differentiates, through the strength of their divergence or convergence, the number sets infiniteness. The algebraic inverse of a *q*-exponential is the *Q*-exponential, *Q* = 2 − *q*. The functional inverse of a *q*-exponential is the *q*-logarithm. Previously [16], we had identified four different size-rank universality classes, worked out their specific nonlinear maps, indicated their tangency features, and found quantitative agreement with real data. Two of these correspond to *α* = *z* = *q* → ∞ (exponential and Gaussian *P*(*N*)) and the other two with *α* = *z* = *q* = 2 (classical Zipf law when *P*(*N*) ∼ 1/*N*^2^) and *α* = *z* = *q* = 1 (hyperbolic *P*(*N*) ∼ 1/*N*). Here we have found that these classes correspond, respectively, to the Factorial numbers (*q* → ∞), the Natural numbers (*q* = 2), and the Fibonacci numbers (*q* = 1).

For iterated maps in the interval, as it is the case here, trajectories can be initiated within a real number set of dimension one. After an infinite number of iterations the attractor can leave this dimension still equal to one or reduce it to a dimension less than one or even make it vanish as its lower limit. We call this dimension the contraction dimension [14], and in the case of the fixed-point map f*(*x*) it is given by *Z* = 2 − *z*, *z* < 2 and *Z* = 0, *z* > 2 (or *α*′ = 2 − *α*, *α* < 2 and *α*′ = 0, *α* > 2, in the notation for the rank functions *N*(*k*) and *F*(*k*′)). As we have seen, when *z* = *Z* = 1 the map is a cusp formed by two lines. For a special value of the slopes and initial conditions the trajectories of this map approximate the Fibonacci numbers and their reciprocals, and the series of the latter converges. The rank functions *N*(*k*) and *F*(*k*′) for this case differ the most, as they follow ordinary exponential and logarithmic functional forms, respectively. When *z* grows above unity, the contraction dimension is *Z* < 1, the map exhibits first a sharp cusp and then, when closer to *z* = 2, the cusp rounds and separates from the identity line (see Fig 6). The rank functions *N*(*k*) and *F*(*k*′) differ less, as both functions follow power-law decay. The contraction dimension first vanishes, *Z* = 0, when *z* = 2, the map (for *Z*) becomes a line parallel to the identity, and for *u* = *x*_0_ = 1 the trajectory generates the Natural numbers. Whereas for the map for *z* = 2 with *u* = 1 the sum of positions of the trajectory initiated at *x*_0_ = −1 generates the (negative) Harmonic numbers. The series of Natural number reciprocals diverges. The rank functions *N*(*k*) and *F*(*k*′) exhibit both the same power law decay, that for the classical Zipf law. When *z* > 2 the map displays a tangent bifurcation with sharper departures from the identity line as *z* increases. The contraction dimension remains *Z* = 0 for al *z* > 2. The rank functions *N*(*k*) and *F*(*k*′) exhibit both power law decay but now the deformed exponential followed by *N*(*k*) decays more slowly than its inverse, the deformed logarithm followed by *F*(*k*′). When *z* → ∞ tangency shifts to *x* → ∞ and, as shown, trajectories can be tuned to approximate the Factorial numbers via Stirling approximation. Again the rank functions *N*(*k*) and *F*(*k*′) differ the most but now ordinary exponential (*F*(*k*′)) and logarithmic (*N*(*k*)) functional forms have been exchanged.

The trajectories *x*_*t*_ with *x*_0_ > 0 from f*(*x*) scape form *x* = 0, while those of their (minus) reciprocals −1/*x*_*t*_ approach *x* = 0, at rates dependent on the nonlinearity *z* > 1. With choice values of *z* we have tuned the spacing out of consecutive *x*_*t*_ (by adjusting the remaining parameters) to reproduce (exactly or asymptotically) the Factorial, Natural and Fibonacci number sets. A measure of the scape rate to infinity is the convergence or divergence of the series of number reciprocals. We found that the series diverges for *z* = 2 (and presumably other *z* < 2 with contraction dimension *Z* ≠ 0), while there is series convergence for all *z* > 2. This borderline value *z* = 2 is similar to the divergence or convergence of the second moment distributions of independent events and of the borderline dimension of critical exponents in continuous phase transitions. We looked for the presence of logarithmic corrections that appear under these circumstances and found that the set of Prime numbers can be accommodated (via the known logarithmic integral function bound) at such boundary in our formalism, signalling the edge for series convergence. We looked at real data to illustrate this circumstance and analyzed the case of earthquake magnitudes as possible candidate. The outcome of our exploration offered a positive answer.

The action of ranking data is performed by computing the (complementary) cumulative distribution *F*(*k*′) from the parent distribution *P*(*N*), and the calculation of the quantile *N*(*k*) involves determining the inverse function of *F*(*k*′) [15]. This task that eliminates existing correlations in the original sample data sets, observable, for instance, when ordered in consecutive time of occurrences or other real circumstance. Thus the action of ranking renders independent outcomes, much like those for the known central limit distributions.

## References

[1] Gutenberg B. Richter C.F. *Seismicity of the Earth and Associated Phenomena*. 2nd ed. Princeton, N.J.: Princeton University Press, 1954.

[2] *Gutenberg-Richter law*. 2021. https://en.wikipedia.org/wiki/Gutenberg-Richter_law.

[3] Zipf G.K. *Human Behavior and the Principle of Least Effort*. Cambridge: Addison-Wesley, 1949.

[4] *Zipf’s law*. http://en.wikipedia.org/wiki/Zipf’s_law.

[5] Benford F. “The law of anomalous numbers”. In: *Proc Am Phil Soc* 78 (1938). [Accessed April 22, 2021], pp. 551–572. http://www.jstor.org/stable/984802.

[6] *Benford’s law*. http://en.wikipedia.org/wiki/Benford’s_law.

[7] Newman M.E.J. “Power laws, Pareto distributions and Zipf’s law”. In: *Contemporary Physics* 46.5 (2005), pp. 323–351. doi: 10.1080/00107510500052444

[8] “To Honor G.K. Zipf”. In: *Glottometrics* 3,4,5 (2002). ISSN: 1617-8351. http://www.ram-verlag.eu/journals-e-journals/glottometrics/.

[9] Kawamura K. Hatano N. “Universality of Zipf’s law”. In: *J. Phys. Soc. Jpn*. 71 (2002), pp. 1211–1213. doi: 10.1143/JPSJ.71.1211

[10] Altamirano C. Robledo A. “Generalized thermodynamics underlying the laws of Zipf and Benford”. In: International Conference on Complex Sciences. Springer. 2009, pp. 2232–2237.

[11] Altamirano C. Robledo A. “Possible thermodynamic structure underlying the laws of Zipf and Benford”. In: *Eur Phys J B* 81.3 (2011), pp. 345–351. doi: 10.1140/epjb/e2011-10968-5

[12] Robledo A. “Laws of Zipf and Benford, intermittency, and critical fluctu-ations”. In: *Chinese Sci Bull* 56.34 (2011), pp. 3643–3648. doi: 10.1007/s11434-011-4827-y

[13] Yalcin C, Robledo A, Gell-Mann M. “Incidence of *q* statistics in rank distributions”. In: *Proceedings of the National Academy of Sciences* 111.39 (2014), pp. 14082–14087. doi: 10.1073/pnas.1412093111 25189773 PMC4191817

[14] Yalcin G.C. Velarde C. Robledo A. “Entropies for severely contracted configuration space”. In: *Heliyon* 1.3 (2015), e00045. doi: 10.1016/j.heliyon.2015.e00045 27441229 PMC4945624

[15] Velarde C. Robledo A. “Rank distributions: Frequency vs. magnitude”. In: PLOS One 12.10 (2017). Ed. by Sanjuán Miguel A. F., e0186015. ISSN: 1932–6203. doi: 10.1371/journal.pone.0186015 28982160 PMC5628998

[16] Velarde C. Robledo A. “Dynamical analogues of rank distributions”. In: *PLOS One* 14.2 (Feb. 2019), pp. 1–15. doi: 10.1371/journal.pone.0211226 30716119 PMC6361506

[17] Pietronero L. et al. “The uneven distribution of numbers in nature.” In: *Physica A: Statistical Mechanics and its Applications* 293 (2001), p. 297. doi: 10.1016/S0378-4371(00)00633-6

[18] Hu B. Rudnick J. “Exact solutions to the Feigenbaum renormalization-group equations for intermittency”. In: *Physical Review Letters* 48 (1982), pp. 1645–1648. doi: 10.1103/PhysRevLett.48.1645

[19] Fisher M.E. “Renormalization group theory: Its basis and formulation in statistical physics”. In: *Rev. Mod. Phys*. 70 (1998), pp. 653–681. doi: 10.1103/RevModPhys.70.653

[20] Schuster H.G. Deterministic chaos. An Introduction. 2nd Edition. Weinheim, Germany: VCH Publishers, 1988.

[21] Logarithmic Integral function. https://en.wikipedia.org/wiki/Logarithmic_integral_function.

[22] Fibonacci number. https://en.wikipedia.org/wiki/Fibonacci_number.

[23] SCEDC (2013): Southern California Earthquake Center. 10.7909/C3WD3xH1

